# Controlling single-molecule junction conductance by molecular interactions

**DOI:** 10.1038/srep11796

**Published:** 2015-07-02

**Authors:** Y. Kitaguchi, S. Habuka, H. Okuyama, S. Hatta, T. Aruga, T. Frederiksen, M. Paulsson, H. Ueba

**Affiliations:** 1 Department of Chemistry, Graduate School of Science, Kyoto University, Kyoto 606-8502, Japan; 2 Donostia International Physics Center (DIPC), 20018 San Sebastián, Spain; 3 IKERBASQUE, Basque Foundation for Science, E-48013, Bilbao, Spain; 4 School of Computer Science, Physics and Mathematics, Linnaeus University, 391 82 Kalmar, Sweden; 5 Division of Nano and New Functional Materials Science, Graduate School of Science and Engineering, University of Toyama, Toyama 930-8555, Japan

## Abstract

For the rational design of single-molecular electronic devices, it is essential to understand environmental effects on the electronic properties of a working molecule. Here we investigate the impact of molecular interactions on the single-molecule conductance by accurately positioning individual molecules on the electrode. To achieve reproducible and precise conductivity measurements, we utilize relatively weak *π*-bonding between a phenoxy molecule and a STM-tip to form and cleave one contact to the molecule. The anchoring to the other electrode is kept stable using a chalcogen atom with strong bonding to a Cu(110) substrate. These non-destructive measurements permit us to investigate the variation in single-molecule conductance under different but controlled environmental conditions. Combined with density functional theory calculations, we clarify the role of the electrostatic field in the environmental effect that influences the molecular level alignment.

Electrical properties of single molecules have attracted much attention due to their potential use as basic components of electronics in the limits of miniaturization^1^,^2^,^3^,^4^,^5^. To this end, significant experimental efforts have been devoted to make molecular junctions and probe their electrical properties. To precisely measure the conductance through a molecule, reliable connections of the metal probes to the molecule must be made^6^. With scanning tunnelling microscope (STM) junctions, one can bridge a “target” molecule between the tip and substrate and reliably study the conductance through it^7^,^8^. Haiss *et al.* successfully picked up one end of dithiol molecules with an STM tip and studied electronic transport as a function of the molecular tilt angle^9^. Such a lift-up control of a single molecule with the STM tip has been used to make molecular junctions of definite geometry^10^,^11^,^12^,^13^,^14^,^15^.

Another characteristics of STM is that individual atoms and molecules can be manipulated on the surface with atomic-scale precision^16^, which was used to investigate and control the interaction between them^17^,^18^,^19^. By accurate positioning of surrounding molecules on the surface, intermolecular effects have been demonstrated on Kondo resonances^20^ and hydrogen transfer reactions^21^. Combined with the manipulation technique, the STM could be used to investigate the effect of surrounding molecules on the junction conductance, known as environmental chemical gating^22^,^23^,^24^,^25^, at an atomistic level. To make good use of these advantages of STM, however, it is essential to preserve an identical tip-molecule contact geometry during repeated junction formation, which then enables one to compare the conductance through a single molecule on the surface in different arrangements and orientations of neighboring molecules.

Here we present such a non-destructive contact that uses a phenyl ring to reversibly connect the molecule to the tip electrode. In a prior work^26^, some of us studied the adsorption of a phenoxy molecule on Cu(110) and found that it is bonded to the surface via an oxygen atom in a nearly flat configuration. When the STM tip is gradually approached to such a flat-lying phenoxy molecule on the surface at one point, the molecule flips up and makes contact to the tip apex while remaining anchored to the Cu surface via the oxygen atom, thus forming a molecular junction between the two electrodes. Because the tip-phenyl interaction is relatively weak (*π*-bonding) as compared to that between molecule and substrate (covalent bonding), retraction of the tip causes cleavage at the same tip-phenyl interface, releasing the molecule to the original position on the surface without any perturbation of the tip apex. Thus, repeated switching of a phenoxy junction is feasible with this setup. This enables us to investigate the molecular conductance with unprecedented precision and to compare the conductance of a junction in different prearranged environments to reveal the impact of surrounding molecules.

## Results

### Controlled switching of a phenoxy molecule

**Figure 1:**
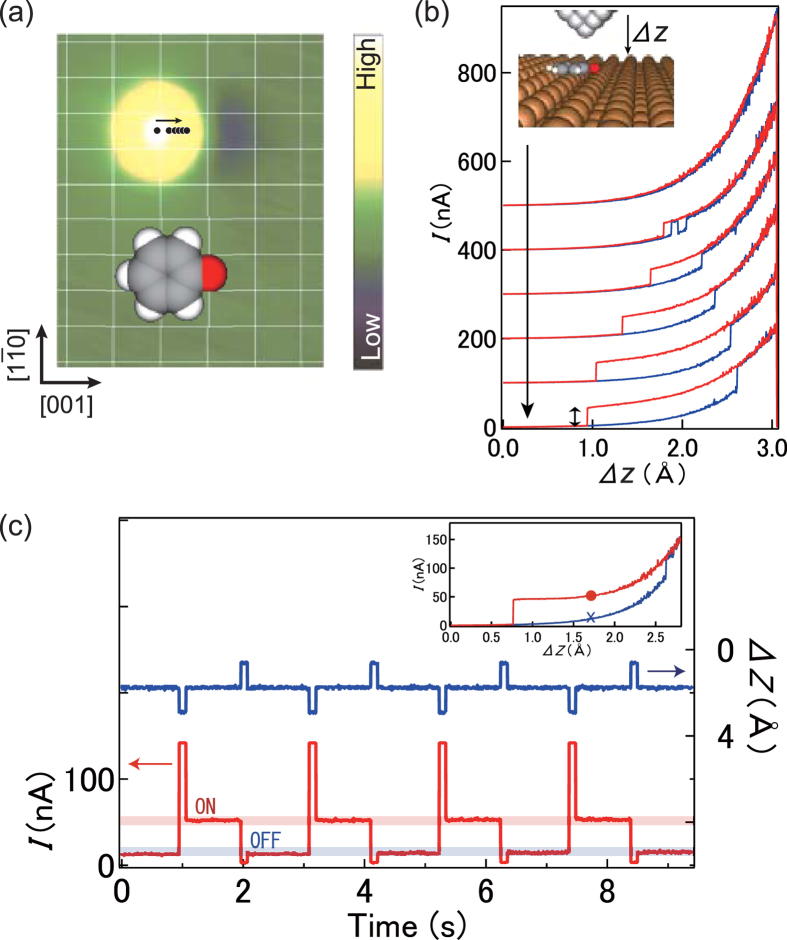
STM images of phenoxy molecules on Cu(110) and controlled switching of the molecular junction. (**a**) An STM image of a phenoxy molecule on Cu(110) with schematic illustration. A pair of protrusion and depression associated with the phenyl ring and oxygen atom, respectively. The white grid lines indicate the lattice of Cu(110). The image size is 19 × 24 Å^2^. (**b**) The tunnel current during the approach (blue curves) and retraction (red curves) of the tip along the surface normal. The curves were recorded at various lateral positions shown in (**a**), and vertically offset for clarity. The origin of the abscissa (Δ*z* = 0) is the initial tip height corresponding to *V* = 50 mV and *I* = 1 nA over the protrusion. (**c**) Repeated switching of the molecular junction by using the conductance hysteresis shown in the inset. The tip was positioned at Δ*z* = 1.7 Å (cross in the inset), and then temporally approached and removed out of the hysteresis region, which switches the junction to the ‘on’ (dot) and ‘off’ (cross) states, respectively.

The STM image of a phenoxy molecule on Cu(110) shows a pair formed by a protrusion and a smaller depression (Fig. 1a), reflecting the density of states at the phenyl ring and oxygen atom, respectively. The white lines show the lattice of surface Cu atoms, with the crossing points indicating the atomic positions. It was revealed that the molecule is nearly flat on the surface and bonded to the short-bridge site via the oxygen atom^26^, as schematically shown in the inset to Fig. 1a.

The phenoxy molecule can be lifted up to the tip and released reversibly by controlling the tip-surface distance. As described below, we prepared the tip apex by coating with copper atoms to realize reliable contact to the molecule. The tip was first positioned precisely over the protrusion at the set point corresponding to *I* = 1 nA at *V* = 50 mV. After the feedback was turned off, the tip was laterally displaced in the [001] direction (dots in Fig. 1a) and then approached to the molecule. Therefore, the initial tip height (Δ*z* = 0) is the same for the curves recorded at different lateral positions.

The tunnel current recorded during the approach and subsequent retraction shows remarkable hysteresis (Fig. 1b). For the bottom curve, for example, the current increases exponentially as a function of Δ*z* according to the tunneling mechanism, and abruptly jumps at Δ*z* = 2.6 Å, suggesting the configurational change of the molecule. The molecule remains in the high-current state as the tip is retracted, until it returns back to the original configuration at Δ*z* = 0.9 Å. We also note that the lateral tip position along [001] critically affects the hysteresis: the closer the tip is positioned to the oxygen binding site, the larger the hysteresis loop.

The high-current state is ascribed to the lift-up of the molecule and the contact formation between the tip and molecule. This is evidenced by the asymptotic behavior of the current to a finite value as the tip is retracted; if a vacuum gap existed in the junction, the current would decay exponentially to zero. After the complete retraction of the tip, the molecule always returns to the original position as confirmed by imaging the area, suggesting that the oxygen atom acts as an anchor to the surface and the phenyl ring is attached (detached) to (from) the tip depending on the tip displacement.

It was previously predicted that a Cu-terminated tip interacts strongly with a benzene molecule, and thus can pick up the molecule from a Cu(110) surface^27^. A C_60_ molecule was also picked up by the STM tip covered with copper^28^,^29^ or gold^28^,^30^, indicating that phenyl or *π*-conjugated species are prone to attractive interaction with a metallic tip. The important role of *π*-metal interactions in single-molecule electron transport was also demonstrated in the break-junction experiments^31^,^32^. The origin of the preferential interaction of the phenyl ring with low-coordinated Cu atom on the tip was argued in terms of organometallics^27^. The phenyl ring is bonded to Cu via rehybridization of *π* orbitals (*π*-bond interaction)^33^,^34^, realizing the stable contact of the molecule to the Cu-terminated tip.

**Figure 2:**
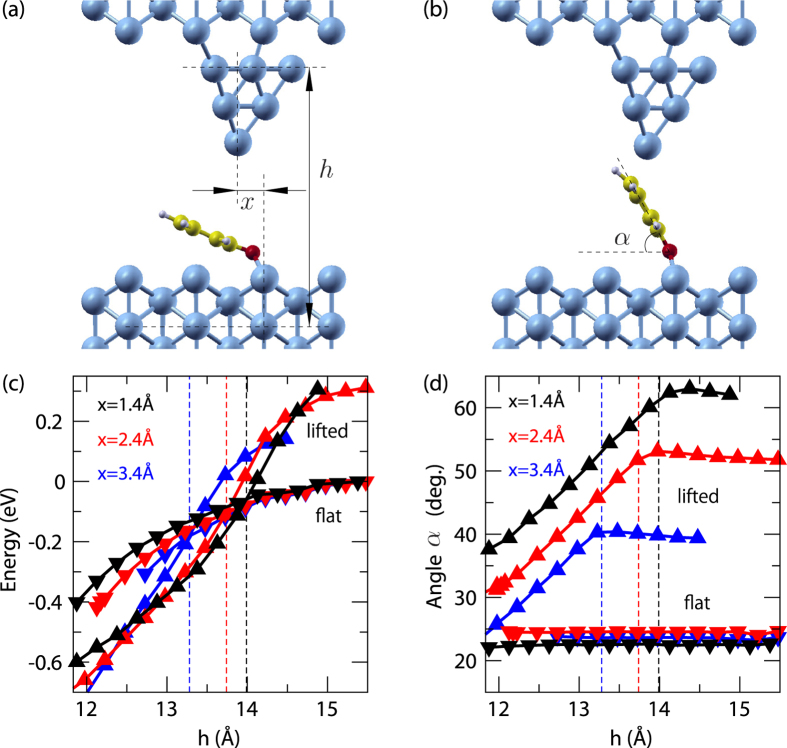
Structure and stability diagram for phenoxy switching. (**a**) Flat and (**b**) lifted configurations of the phenoxy molecule adsorbed on Cu(110) along the [001] direction (tip height *h* = 13.9 Å; lateral tip position *x* = 1.4 Å with respect to the Cu bridge site). The tip height *h* is related with Δ*z* as *h* = *z*_0_ − Δ*z*, where *z*_0_ is the initial tip height. (**c**) Total energy differences and (**d**) molecular tilt angle *α* as a function of tip position. Vertical dashed lines indicate the tip height where flat and lifted configurations are energetically equal. (blue data: *x* = 3.4 Å, red: *x* = 2.4 Å, black: *x* = 1.4 Å; down-triangles: molecule flat, up-triangles: molecule lifted up).

To further understand the experimental observations, the potential energy (Fig. 2c) and molecular tilt angle (Fig. 2d) were calculated for the flat (Fig. 2a) and lifted (Fig. 2b) configurations as a function of the tip height and lateral position. When the tip is far from the surface (large *h*, Fig. 2c) the flat configuration (down-triangles) is more stable. As the tip is approached to the molecule, the lifted molecular conformation (up-triangles) eventually becomes energetically more favorable. Around this crossover (dashed vertical line), an energetic barrier separates the two conformations (Supplementary Fig. 1) thus explaining the observed switching behavior and hysteresis in the *I*-Δ*z* curves. The crossover between flat and lifted configurations is found to depend on the lateral position of the tip along [001] (Fig. 2c). When the tip is positioned laterally closer toward the oxygen bonding site, the lifted configuration is stable at larger tip heights, resulting in the longer plateau of the current as the tip is retracted (Fig. 1b). We postulate that the phenyl ring is sliding on the tip during retraction, giving rise to the smooth plateau.

Formation of tip-molecule contacts via a conformational change of the molecule was previously conducted, mainly to investigate the junction conductance^9^,^10^,^11^,^12^,^13^,^14^,^15^. But in these junctions the covalent bonding between the tip and molecule provided too strong wiring to be controllable. Instead, we utilize here a relatively weak *π*-bond interaction at the tip-molecule interface while the molecule remains firmly anchored to the substrate via the strong chalcogen (covalent) bond. This asymmetric coupling to the electrodes enables robust and controlled switching of the molecular junction with perfect reproducibility. This actuation via displacement of the tip is demonstrated in Fig. 1c. The tip is approached to Δ*z* = 1.7 Å (*I* = 10 nA), retaining the junction in the ‘off’ state (the cross in the inset). By temporarily approaching the tip by 1.0 Å, the junction switches to the ‘on’ state and the current increases to *I* = 50 nA (the dot in the inset). Subsequently, the junction is switched off by retracting temporarily the tip. The on/off conductance ratio depends on the initial Δ*z* position: at Δ*z* = 1.7 Å is found to be 5.0. The ratio can be increased further by operation at a smaller Δ*z*. The tip-molecule contact geometry remains identical between the repeated switching, enabling uniform on/off cycles.

### Environmental impact on molecular conductance

**Figure 3:**
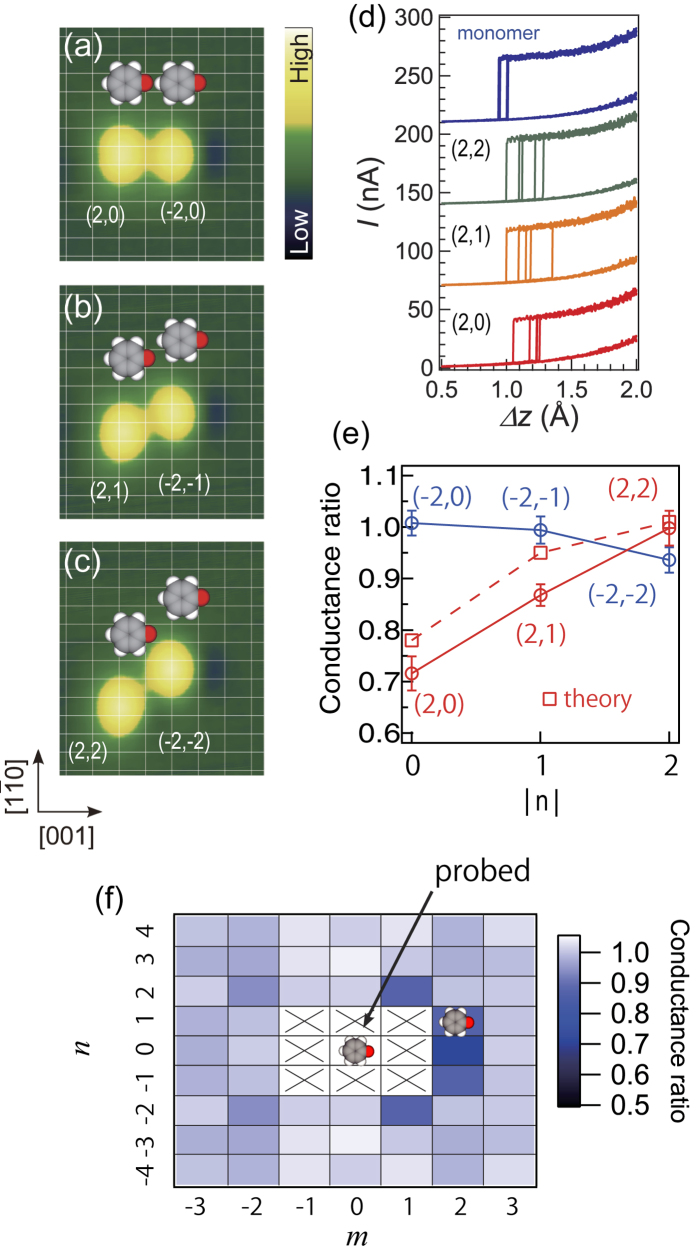
The effect of intermolecular coupling to the molecular conductance. (**a**) STM image of a phenoxy dimer arranged along [001] with a separation of two atomic distance (2*b*_0_). (**b**) The left molecule is moved in the [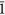
10] direction by one atomic distance (*a*_0_ = 2.56 Å). (**c**) The molecule is further displaced. The molecular configurations are represented by (*m*,*n*), indicating the position of neighboring molecule relative to the probed molecule. The image sizes are 28 × 32 Å^2^. (**d**) Typical current plateau for the probed molecule in the (2,2), (2,1), and (2,0) configurations, together with that for the isolated monomer. The curves were obtained with the same tip apex. (**e**) The conductance data for the (2,2), (2,1), (2,0), (−2,−2), (−2,−1), and (−2,0) configurations are shown by circles. The calculated data for the first three are shown by squares. The data are represented by the ratio to that for an isolated molecule, and the error bars are the standard deviation of the collected data for different tips and molecules. The calculated data shown are the average values for *h* = 13.9 Å and *h* = 14.4 Å (*x* = 1.4 Å, Supplementary Fig. 5). (**f**) The position dependence of the molecular conductance displayed by the color scale. The probed molecule is located at the origin with another (perturbing) molecule located at (*mb*_0_,*na*_0_). The conductance is reduced for the configurations of (2,0), (2,±1) and (1,±1) within the experimental uncertainty and converges to that of a monomer as the molecule is brought apart.

The height of the plateau during the tip retraction (Fig. 1b) is associated with the conductance through the molecule. The ‘on’ curves are inherently mixing two electron pathways: the electron tunneling through the molecule and the one through the tip-surface gap. While it is not possible to exactly separate them, we assume that the latter is associated with the current in the ‘off’ state. Thus, we define the molecular conductance as the conductance difference between the ‘on’ and ‘off’ states, which is determined from the current change at the junction cleavage in the *I*-Δ*z* curve (both-ended arrow for the lowest curve in Fig. 1b). The value of the molecular conductance depends on the tip apex, and is distributed at (1.0 ± 0.3) × 10^−2^ G_0_ (Supplementary Fig. 2) for an isolated molecule, where G_0_ = 2*e*^2^/*h* is the quantum of conductance. Because we can repeatedly make and remove the contact to a molecule with the same tip apex, the conductance through a single phenoxy molecule can also be probed as a function of the position of a neighboring phenoxy on the surface as shown in Fig. 3.

The manipulation of a phenoxy molecule is conducted by lifting it up, followed by lateral displacement of the tip along the [1
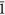
0] direction to the desired position and then release of the molecule (Supplementary Fig. 3). In the following we denote by (*m*,*n*) phenoxy dimer configurations in which the conductance of a probed molecule is characterized in terms of the position of a counterpart molecule at *mb*_0_ and *na*_0_, where *b*_0_ and *a*_0_ are atomic distances on the surface along the [001] and [1
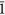
0] direction. For example, the conductance of the left (right) molecule in Fig. 3a—probed with the counterpart located at 2*b*_0_ (−2*b*_0_) along [001] and 0*a*_0_ along [1
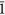
0], respectively—is represented by (2,0) [(−2,0)]. The impact of intermolecular coupling on the conductance for dimer configurations is reported in Fig. 3d,e.

The conductance values for individual molecules are robust between repeated measurements as shown in Fig. 3d, where it is clearly observed that the conductance is reduced for (2,0) and (2,1) as compared to that for an isolated monomer. The (2,0) conductance (lifting up the left molecule in Fig. 3a) is reduced by ~30%, while the (−2,0) conductance (lifting up the right molecule) is nearly equal to that for an isolated monomer (Fig. 3e). This asymmetry arises from the fact that the two molecules are situated in different environments; the molecule adjacent to the phenyl ring (not the oxygen atom) of the neighboring molecule is specifically reduced in the conductance. The (2,1) conductance (lifting up the left molecule in Fig. 3b) is still influenced by the interaction and reduced by ~15% compared to that for an isolated monomer. The conductance for the monomer is almost recovered as the neighbor molecule is positioned farther away from the probed molecule (Fig. 3e). It is noted that the conductance value (the on-off difference) compared at fixed Δ*z* (electrode distance) gives the same result (Supplementary Fig. 4); the environmental effect is robust irrespective of how we define the molecular conductance. This is ascribed to the fact that the on-off difference is not so much dependent on Δ*z*.

The molecular conductance was investigated for dimer configurations in the full two dimensional space (*m*,*n*) as summarized in Fig. 3f. In addition to (2,0) and (2,±1), the conductance for (1,±2) is reduced by ~15%. Further reduction of the intermolecular distance is hampered by repulsive interaction between the molecules (crosses in Fig. 3f). The conductance for other dimer configurations is nearly equal with that for an isolated molecule (without interaction); the influence over ~8 Å distance is too small to be detected. Fig. 3f also clearly shows the anisotropy of the intermolecular effect: the effect is significant only when the counterpart is located at the oxygen side of the probed molecule (configurations with positive *m*).

**Figure 4:**
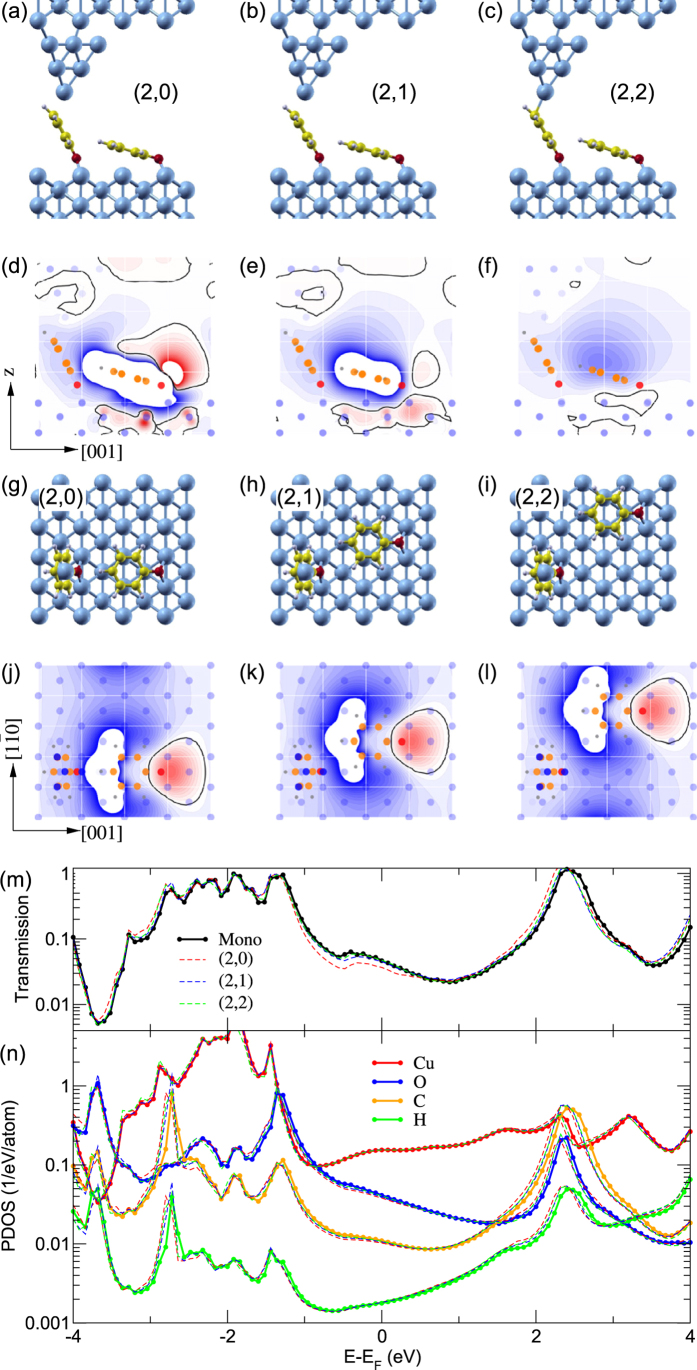
Calculation of the environmental effect for different dimer junction geometries. (**a**–**c**) Side views of (2,0), (2,1), and (2,2) dimer junction geometries with one molecule lifted up by the STM tip. (**d**–**f**) The changes in the electrostatic potential (*δV*) induced by a neighbor phenoxy molecule in the dimer junction geometries. (**g**–**i**) Top views of (2,0), (2,1), and (2,2) dimer junction geometries. (**j**–**l**) Corresponding changes in the electrostatic potential. The tip height *h* = 13.9 Å and lateral position *x* = 1.4 Å. The projections are onto planes through the center of the conducting molecule (to the left in each visualization). Contour lines are separated by 25 meV. Blue (red) areas indicate a positive (negative) change in potential and the thick contour line the zero-value. Atomic positions are shown as colored circles (blue: Cu, gray: H, orange: C, red: O). White lines in (**j**–**l**) indicate the Cu(110) lattice. (m) Transmission and (n) projected density of states (PDOS) onto the Cu apex atom as well as onto the atoms in the current-carrying phenoxy molecule [thick lines: monomer; red dashed: (2,0); blue: (2,1); green: (2,2) configurations]. Tiny shifts of the resonances to lower energies are observed in the dimer configurations. For the (2,0)-dimer the transmission probability near the Fermi level *T*(*E*_*F*_), and hence the low-bias conductance, is lowered with respect to the other configurations.

The environmental effect on the junction conductance is also captured by our simulations (squares in Fig. 3e) for dimer structures (Fig. 4), in good agreement with the experimental data. The transmission functions (Fig. 4m) reveal that electrons are tunneling through the gap of molecular states, observed as well-defined resonances in the the projected density of states (PDOS) onto the C and H atoms (Fig. 4n). The highest occupied molecular orbital (HOMO), more than 1 eV below the Fermi level *E*_*F*_ in our calculations, hybridizes significantly with *sd*-states of Cu leading to multiple peaks in that energy range. The lowest unoccupied molecular orbital (LUMO) on the other hand, about 2–3 eV above *E*_*F*_, retains a rather simple lineshape. The observed reduction of the junction conductance by a neighbor molecule can be traced back to electrostatic gating of the probed molecule in contact with the tip. Figure 4d–f,j–l and Supplementary Fig. 5 display the induced electrostatic potential by adding the second molecule to the cell, defined as the potential difference *δV*(*x*,*y*,*z*) = *V*_*A*_(*x*,*y*,*z*) − *V*_*B*_(*x*,*y*,*z*), where *A* (*B*) corresponds to a self-consistent calculation including (excluding) the flat neighbouring phenoxy molecule. Fig. 4d,j show projections onto planes through the center of the conducting molecule in a (2,0) dimer configuration. In the region of the probed molecule (lower left parts) an electrostatic shift of the order 100 mV is found. The shift gradually diminishes for the (2,1) dimer (Fig. 4e,k) and the (2,2) dimer (Fig. 4f,l), respectively. The reduced dimer conductance can thus be understood as a consequence of an electrostatic downshift of the molecular orbitals with respect to the Fermi level (Supplementary Fig. 6). The electrostatic effect can also be rationalized by just considering the induced electrostatic potential from an isolated flat-lying phenoxy molecule on Cu(110) (Supplementary Fig. 7). Further, the strong asymmetry in the dipolar field explains why the probed molecule is essentially only affected when the counterpart is located at the oxygen side as mentioned above.

**Figure 5:**
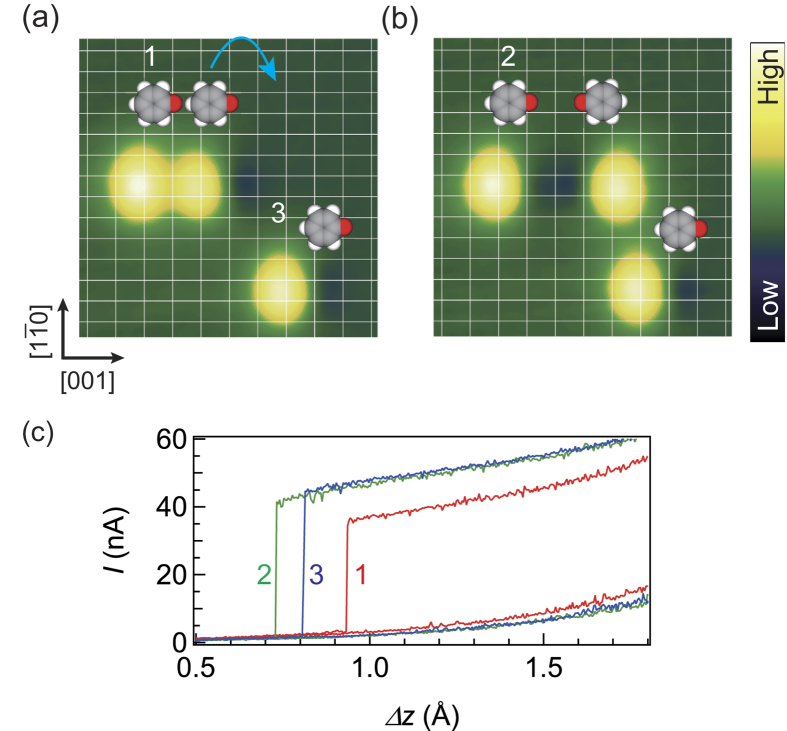
The control of single-molecule junction conductance through the orientation of a neighbor molecule. (**a**) STM images of phenoxy dimer and monomer. (**b**) The same area as (**a**), whereby one molecule is flipped in the opposite orientation. The image sizes are 38 × 38 Å^2^. (**c**) Typical current plateau for the probed molecules 1–3 in (**a**) and (**b**). The curves were obtained with the same tip apex.

We demonstrate above that the junction conductance can be tuned by changing the position of the neighbor molecule. Alternatively, it is feasible to toggle the conductance between two levels by simply changing its orientation. In the dimer (2,0) configuration (‘1’ in Fig. 5a), the junction conductance is reduced by ~30% (Fig. 5c) as compared to that for an isolated molecule (‘3’ in Fig. 5a). By flipping the adjacent molecule, we can remove the interaction and thus recover the original conductance value (‘2’ in Fig. 5b). The flip of the molecule is conducted by lifting it up, followed by lateral displacement of the tip in the [001] direction by ~8 Å, resulting in the release of the molecule to the opposite orientation. This illustrates electrostatic control or “gating” of the junction conductance through the conformation change of an adjacent molecule. While it is challenging to integrate a gate electrode in the STM set-up, molecular interaction could be potentially used to control the junction conductance through the shift of the electronic level of the molecule.

### Effect of molecular density

**Figure 6:**
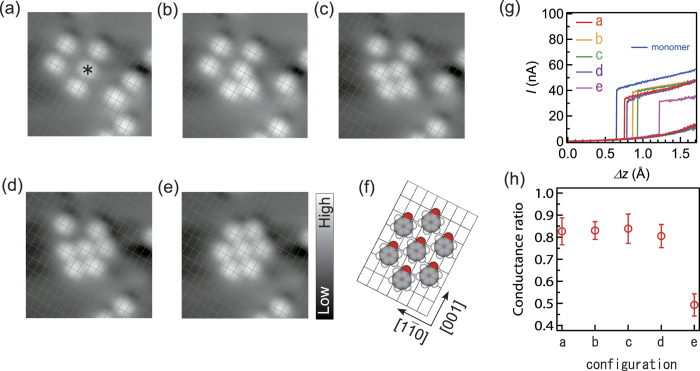
The dependence of the molecular conductance on the density of the molecules. (**a**) Three phenoxy molecules along the [001] direction. The molecular conductance was monitored for the center molecule (asterisk) as the surrounding molecules are sequentially attached to the island [(**b**–**e**)]. The image sizes are 42 × 42 Å^2^. (**f**) The schematic structure of the c(2 × 4) island in (**e**). (**g**) Typical *I*-Δ*z* curves for the center molecule in the configurations of (**a**–**e**). The curves were obtained with the same tip apex. (**h**) The molecular conductance data for the center molecule. The conductance is represented by the ratio to that for an isolated monomer. The conductance is ~0.8 in the chain [(**a**)] because of the intrachain electrostatic effect, and decreases to ~0.5 when the molecules form the dense island [(**e**)].

Finally, we also explore the molecular conductance as a function of the density of the molecules. First, three phenoxy molecules are arranged in a chain along the [001] direction (Fig. 6a). The conductance of the center molecule (asterisk) is monitored and determined to be ~0.8 with respect to that of the monomer. This is ascribed to the intermolecular effect in the chain, as described above. Then we manipulate surrounding molecules one by one and build an island composed of seven phenoxy molecules (Fig. 6b–e). The interchain distance is 2*a*_0_ in the island, and the molecules form local c(2 × 4) superstructure which is the most dense phase of this adsorption system (Fig. 7f)^35^. In the build-up process, the conductance of the center molecule was monitored (Fig. 6h). The conductance is the average determined from five cycles of *I*-Δ*z* curves (Supplementary Fig. 8). The typical *I*-Δ*z* curves are shown in Fig. 6g. The arrangement of neighboring molecules at the (−1,−2) (Fig. 6b), (−1,2) (Fig. 6c), and (1,−2) (Fig. 6d) positions causes only negligible influence on the conductance of the center molecule. On the other hand, the formation of the c(2 × 4) island with the molecule at the (1,2) position (Fig. 6e) causes dramatic reduction of the conductance by ~50%. This indicates that the dense structure is responsible for the reduction in the conductance. The molecule at the (1,−2) or (1,2) position causes a negative electrostatic shift of the center molecule (Fig. 3f). We suggest that the occupation of both positions by two molecules enhances the electrostatic effect, explaining the drastic reduction in the conductance of the center molecule.

## Discussion

In previous works the molecular conductance was thoroughly investigated by break-junction experiments as a function of the molecular structure^36^,^37^,^38^ and anchoring groups^39^,^40^,^41^. The molecular bonding site and angle to the electrode play significant roles in determining the molecular conductance. Another important variable is the density of molecules on the electrode, i.e., how surrounding molecules affect the transport property of a probed molecule^42^. The effect of solvent environment on the single-molecule conductance through electrostatic coupling between the molecules was also revealed^25^. Here, we have pushed the study of these determining factors using STM techniques by demonstrating atomic-scale control over the effect of molecular interaction and molecular density on the conductance of single-molecule junctions. Our novel strategy relies on manipulation of adsorbed molecules and reproducible contact formation to achieve well-defined conditions with accurate location of individual molecules with respect to each other. The present work reveals that the conductance through a molecule varies nearly by a factor two depending on its environmental conditions, i.e., whether it is isolated or densely packed, highlighting the critical role of molecular interactions in single-molecule electron transport. We demonstrate that it is feasible to control the junction conductance by switching the configuration of a molecule located nearby.

By utilizing *π*-bonding interaction, we make and remove a molecular contact to a metal electrode without any perturbation to the electrode as well as to the molecule. By controlled switching of the junction repeated conductance measurements with identical molecule-electrode interface can be carried out. The present approach to the molecular junction with a *non-destructive* contact enables precise evaluation of the molecular conductivity, as demonstrated by the detection of environmental effect on the conductance. We envision that our reproducible tip contact could be applied to larger functional molecules with terminal phenyl group used as molecular “alligator clip” to precisely characterize their electronic properties.

## Methods

### A. Experimental

The experiments were performed in an ultra-high vacuum chamber equipped with STM operating at *T* = 4.5 K. The Cu(110) surface was cleaned by repeated cycles of argon ion sputtering and annealing. The surface was exposed to phenol vapor at 300 K, giving rise to partial dehydrogenation to phenoxy groups on the surface^26^. The produced phenoxy molecules favor chain formation along the [001] direction. The STM images were obtained at sample bias *V* = 50 mV and tunnel current *I* = 1 nA. Electrochemically etched tungsten tips were used as an STM probe. The tips were repeatedly and gently touched to the Cu surface to coat them with copper, to prepare Cu-terminated tips for reliable switching^27^. We have seen that ‘sharp’ tips giving high-contrast images are prone to successfully lift up the molecules. It is also important to avoid “molecular” tips for reliable switching. The structure of the tip apex reflects the shape of the *I*-Δ*z* curve. We can routinely optimize the tip apex by repeated touching of the surface so that smooth *I*-Δ*z* curves are obtained as shown in Fig. 1b.

The phenoxy molecule can be displaced along the [1
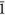
0] direction by STM manipulation (Supplementary Fig. 3). First, the molecule (asterisk in Supplementary Fig. 3a) was lifted up by the tip. Then the tip was displaced to the desired position along the [1
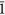
0] direction while the junction was maintained. The tunnel current recorded as a function of the displacement (Supplementary Fig. 3c) shows “saw-tooth” shape, indicating lateral hopping of the molecule. After the manipulation, the molecule was found to be displaced by 4*a*_0_ (Supplementary Fig. 3b). In this way, we can manipulate individual phenoxy molecules along the [1
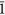
0] direction and form dimers (Fig. 3) or islands (Fig. 6) to investigate the intermolecular effect on the conductance.

### B. Computational

#### Electronic structure methods

The atomic structure and total energy were determined using Kohn-Sham density functional theory (DFT) with a plane-wave basis using VASP^43^,^44^. We used the optPBE-vdW^45^ exchange-correlation functional taking into account dispersion, a 400 Ry energy cutoff, and the Gamma point for Brillouin zone sampling. The supercells contained a single phenoxy molecule adsorbed on a five-layer Cu(110) slab with 4 × 5 periodicity (lattice constant *a* = 3.64 Å) as well as a Cu(111) pyramid mounted on the reverse side (representing the STM tip). The molecule, the two topmost surface layers, and the four Cu apex atoms were relaxed until residual forces were smaller than 0.02 eV/Å. We checked that our results are qualitatively unchanged with respect to calculations with the standard GGA-PBE^46^ exchange-correlation functional (Supplementary Fig. 9) or with a Cu(100)-oriented tip model (Supplementary Fig. 10–11). Molecular dimers were simulated with two molecules in the cell. The geometry of the conducting molecule is only affected little by the presence of the second molecule and the variation in the angle *α* is less than 2° (Supplementary Tab. I).

#### Transport simulations

To simulate electron transport through the phenoxy junctions obtained with VASP we performed calculations with Siesta^47^/TranSiesta^48^ in combination with Inelastica^49^. We used a long-ranged double-*ζ* plus polarization (DZP) basis set for the C, H, O, S and Cu surface layer and tip atoms and a short-ranged single-*ζ* plus polarization (SZP) basis set for the bulk Cu atoms. The GGA-PBE^46^ exchange-correlation functional, a cutoff energy of 200 Ry for the real-space integrations, and a 2 × 2 *k*-point sampling were used for the electronic structure calculations. Projected density of states (PDOS) and electron transmission were converged with respect to *k*-point sampling with Gauss-Kronrod quadrature and 21 × 11 evaluation points^49^. The surface Green’s functions for the bulk electrodes were calculated recursively with an imaginary part of *η* = 0.1 eV added to the energy^49^. The low-bias conductance is derived from the Landauer formula *G* = *G*_0_*T*(*E*_*F*_), where G_0_ = 2*e*^2^/*h* is the conductance quantum and *T*(*E*_*F*_) is the transmission probability for electrons at the Fermi energy *E*_*F*_. The corresponding conductance ratios for the dimers (with respect to the monomer), revealing a suppression of up to 25%, are given in Supplementary Tab. II. We checked that this reduction is not simply due to geometric effects by computing the conductance for a lifted phenoxy monomer also in the configurations corresponding to the (2,0)-, (2,1)-, and (2,2)-dimer geometries (replacing the neighboring molecule with ghost orbitals). The variation here would at most explain up to 4% of the conductance reduction.

## Additional Information

**How to cite this article**: Kitaguchi, Y. *et al.* Controlling single-molecule junction conductance by molecular interactions. *Sci. Rep.* **5**, 11796; doi: 10.1038/srep11796 (2015).

## Supplementary Material

Supplementary Information
