# Supplementary material for: Controlling single-molecule junction conductance by molecular interactions
Source: Sci Rep. 2015 Jul 2;5:11796. doi: 10.1038/srep11796 (PMC4488765; doi:10.1038/srep11796)
Supplement: Supplementary Information [file srep11796-s1.pdf]

Supplementary information: Controlling single-molecule junction conductance by molecular interactions

Y. Kitaguchi,[1] S. Habuka,[1] H. Okuyama,[1*] S. Hatta,[1] T. Aruga,[1] T. Frederiksen,[2,3] M. Paulsson,[4] and H. Ueba[5]

[1]Department of Chemistry, Graduate School of Science, Kyoto University, Kyoto 606-8502, Japan

[2]Donostia International Physics Center (DIPC), 20018 San Sebastián, Spain

[3]IKERBASQUE, Basque Foundation for Science, E-48011, Bilbao, Spain

[4]School of Computer Science, Physics and Mathematics, Linnaeus University, 391 82 Kalmar, Sweden

[5]Division of Nano and New Functional Materials Science, Graduate School of Science and Engineering, University of Toyama, Toyama 930-8555, Japan

*Corresponding author: *hokuyama@kuchem.kyoto-u.ac.jp*

(Dated: May 15, 2015)

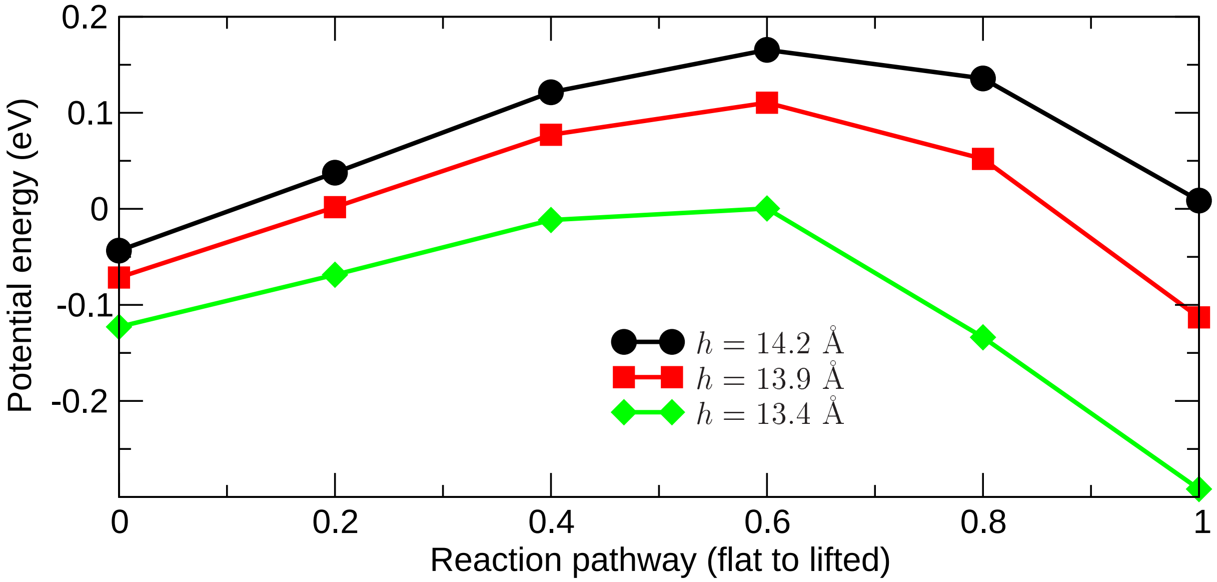

FIG. S1. **Energy barrier for phenoxy switching with the nudged elastic band (NEB) method and the optPBE-vdW functional.** Tip lateral position is set to $x = 1.4$ Å. Around the crossover at $h \approx 14$ Å (where the flat and lifted configurations are energetically equivalent) a barrier of the order 0.2 eV separates the molecular conformations. The common energy reference is the flat configuration with the tip far away from the molecule ($h = 15.4$ Å, cf. Fig. S9).

Tip height (Å)	α_{monomer} °	$\alpha_{(2,0)}$ °	$\alpha_{(2,1)}$ °	$\alpha_{(2,2)}$ °
13.9	60.1	62.1	59.5	60.0
14.4	63.0	65.2	62.4	63.9

TABLE S1. Angle α for a conducting (lifted) phenoxy molecule in either the monomer or one of three dimer configurations $(m,n) = (2,0),(2,1),(2,2)$. Two different tip heights are considered. The lateral tip position is $x = 1.4$ Å.

Tip height (Å)	$G^{\text{PBE}}_{\text{monomer}}$ (G_0)	$R_{(2,0)}$ –	$R_{(2,1)}$ –	$R_{(2,2)}$ –
13.9	0.047	0.79	0.97	1.02
14.4	0.037	0.75	0.93	1.03

TABLE S2. Phenoxy monomer conductance G_{monomer}, dimer conductance ratios $R_{(m,n)} \equiv G_{\text{monomer}}/G_{(m,n)}$ [$G_{(m,n)}$ being the conductance with a neighbor phenoxy molecule located at (m,n)], at two different tip heights. The conducting molecule is in the lifted configuration and the lateral tip position is $x = 1.4$ Å.

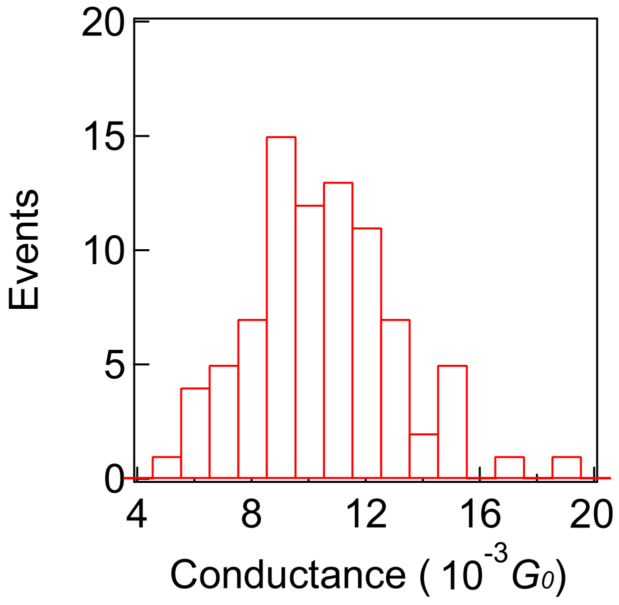

FIG. S2. **The conductance histogram for an isolated phenoxy molecule.** The conductance of the molecule depends on the tip apex. The tip apex was modified by a gentle touch to the surface and the measurement procedure was repeated. The conductance was measured with 84 different tip apexes, giving the distribution of $(1.0\pm0.3)\times10^{-2}$ G_0, where $G_0 = 2e^2/h$ is the quantum of conductance.

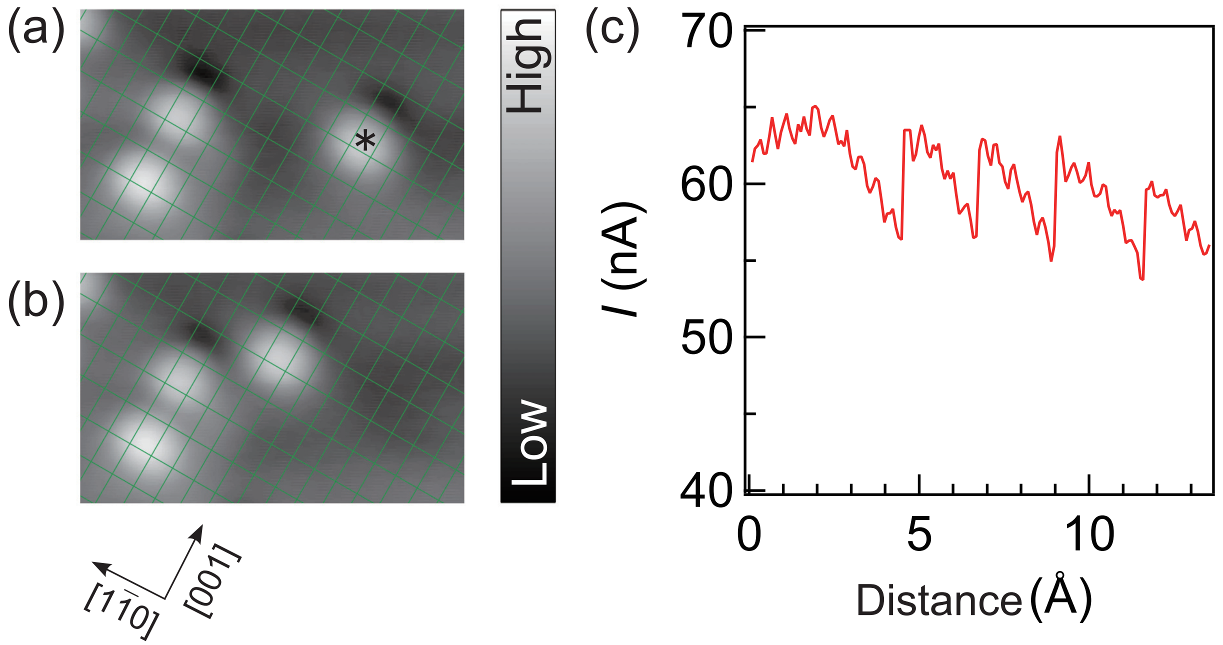

FIG. S3. **The manipulation of a phenoxy molecule with the STM.** (a) Three phenoxy molecules superimposed with the lattice of Cu(110). The right molecule (asterisk) was manipulated by STM in the [1$\bar{1}$0] direction by $4a_0$, as shown in (b). The manipulation was conducted as follows: The tip was positioned over the molecule and lifted up the molecule to form a junction. Then, the tip was laterally moved in the [1$\bar{1}$0] direction while the junction was maintained. The tunnel current recorded during the lateral translation of the tip is shown in (c). The "saw-tooth" shape of the current indicates controlled manipulation of the molecule between the bonding (short-bridge) sites.

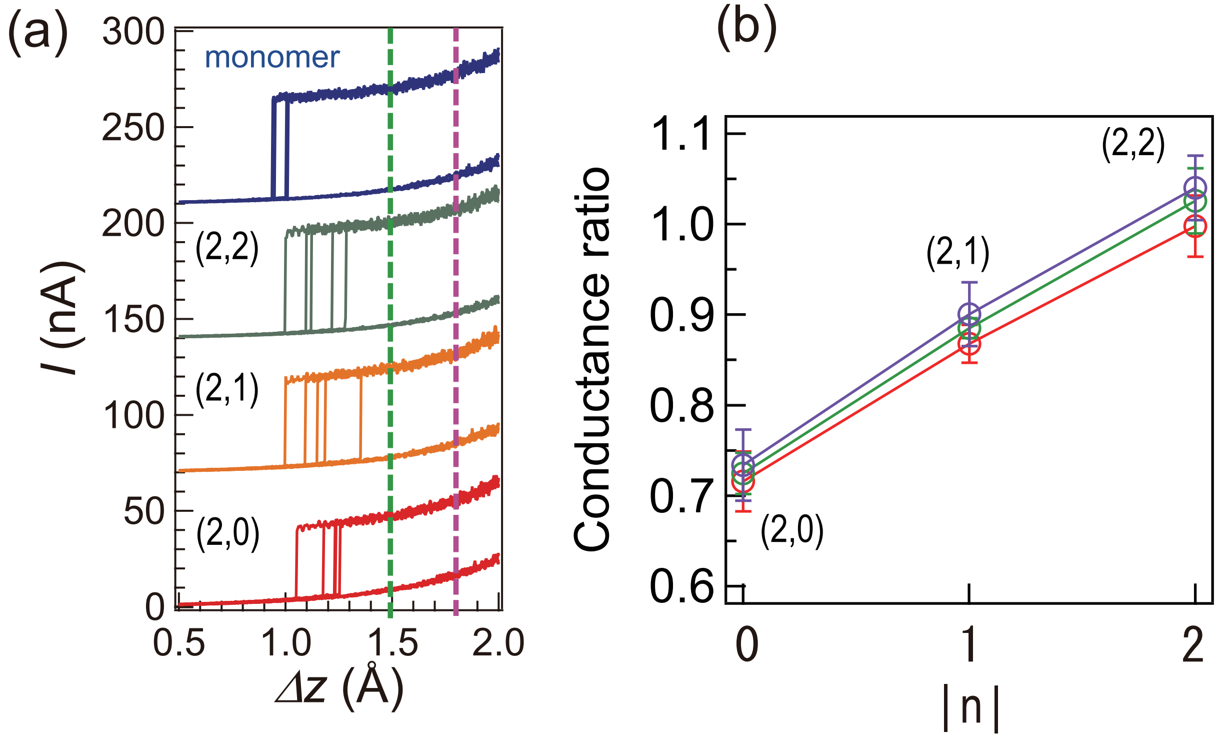

FIG. S4. **Molecular conductance determined from current plateau at the junction rapture or at the same Δz** (a) The I-Δz curves recorded for different environmental species shown in Fig. 3. (b) The red circles represent the molecular conductance determined from the current plateau at the junction rupture (the same as Fig. 3e). On the other hand, the green (violet) circles represent the conductance determined from the difference between the 'on' and 'off' states at Δz=1.5 (1.8) Å [dashed lines in (a)]. The molecular conductance is robust within the experimental uncertainty.

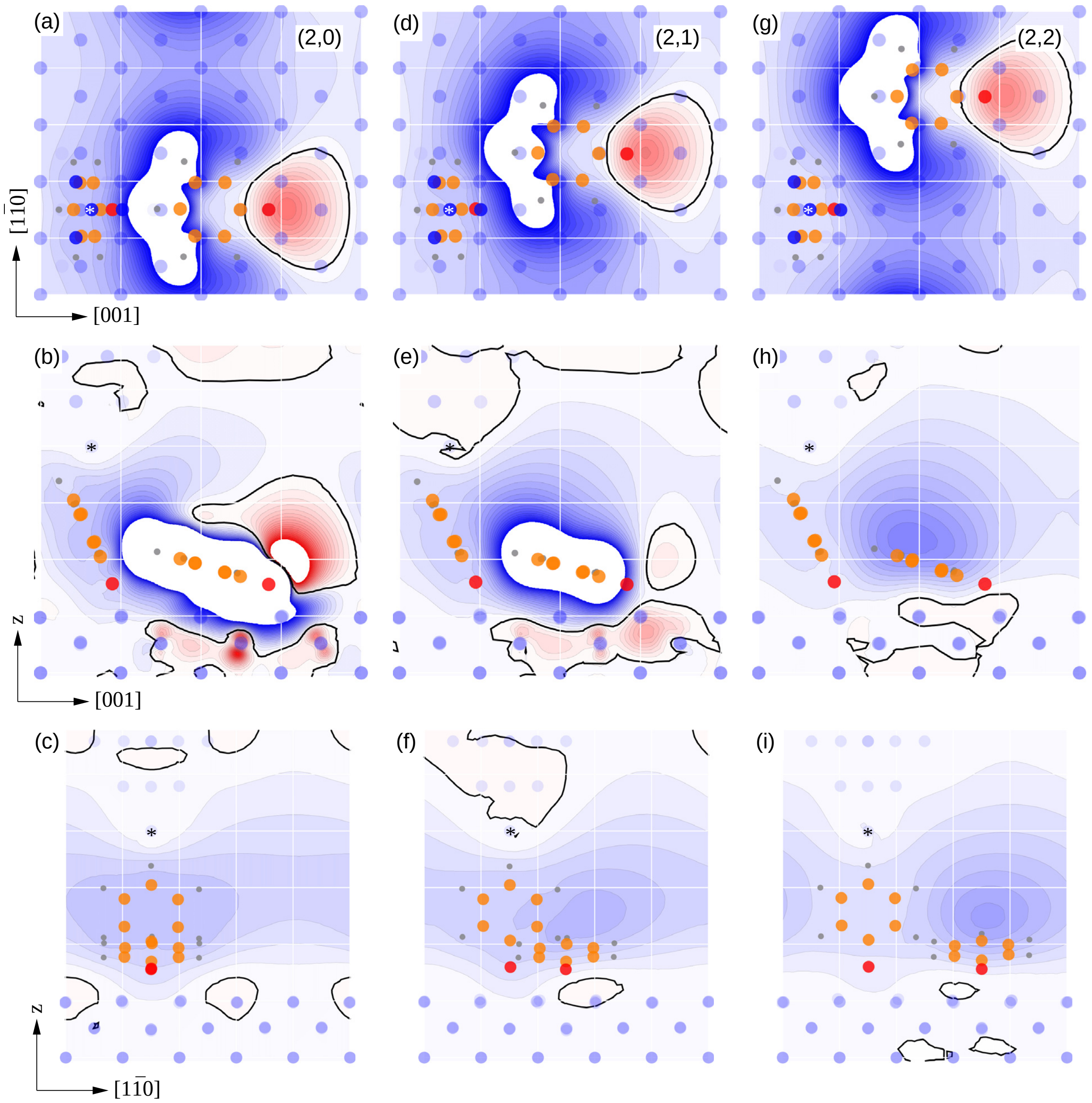

FIG. S5. **The change in the electrostatic potential (δV) induced by a neighbor phenoxy molecule in the junction geometry.** Projections onto planes through the center of the conducting molecule. (a,b,c) (2,0)-configuration, (d,e,f) (2,1)-configuration, and (g,h,i) (2,2)-configuration at tip height $h = 14.4$ Å and lateral position $x = 1.4$ Å. Contour lines are separated by 25 meV. Blue (red) areas indicate a postive (negative) change in the potential and the thick contour line the zero-value. Atomic positions are shown as colored circles (blue: Cu, gray: H, orange: C, red: O). White lines indicate the Cu(110) lattice.

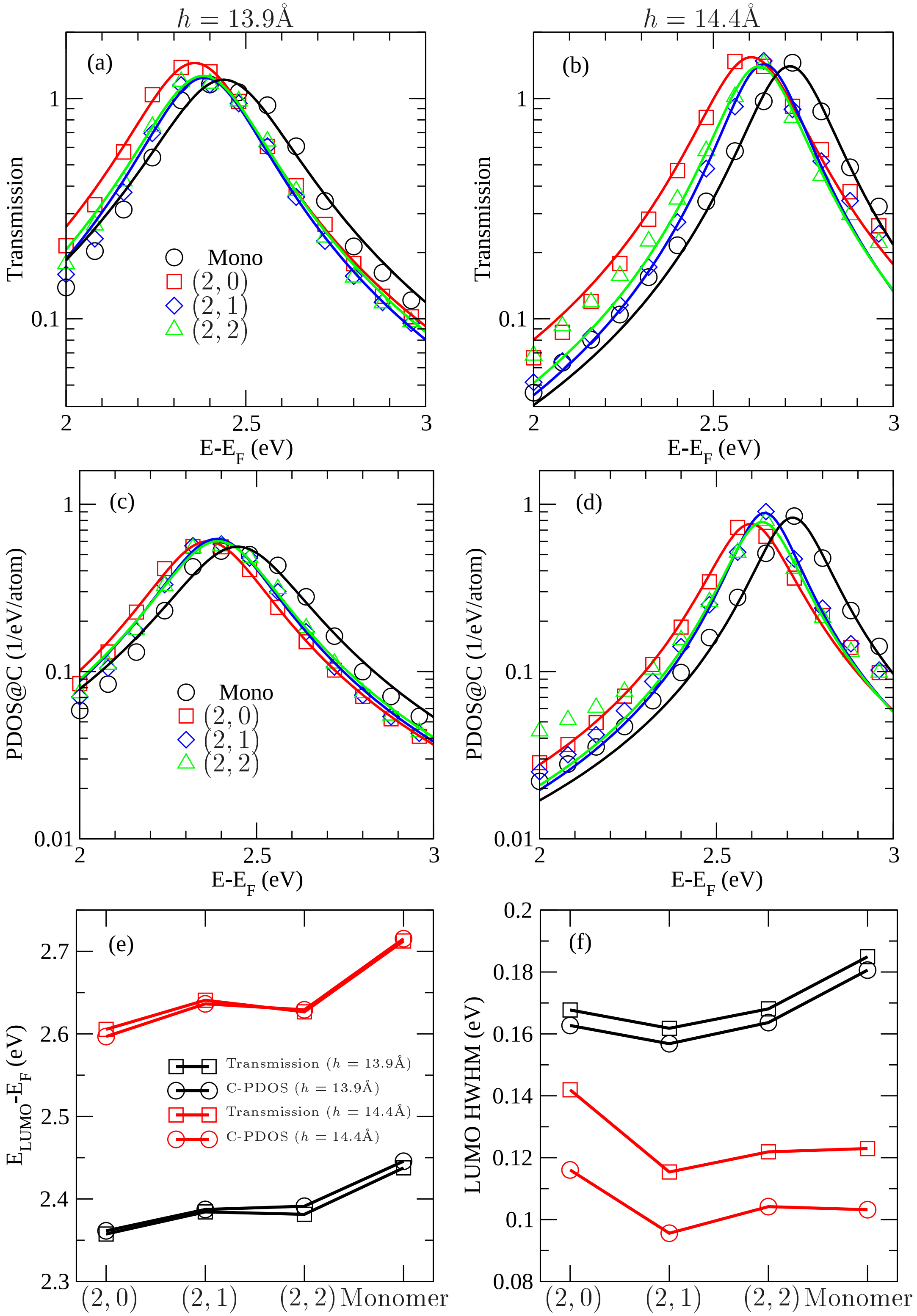

FIG. S6. **Analysis of LUMO position and width for phenoxy dimers.** (a),(b) Lorentzian fits to the transmission $T(E)$ (top row) as well as to the (c),(d) PDOS onto C-atoms of molecule in contact with the tip. (e),(f) The fitted LUMO position and resonance width (half width half max, HWHM) are shown in the bottom row. The tip heights are $h = 13.9$ Å (left column) and $h = 14.4$ Å (right column) and the lateral tip position is $x = 1.4$ Å. Compared with the monomer, a neighboring phenoxy molecule induces a down-shift of the molecular resonances.

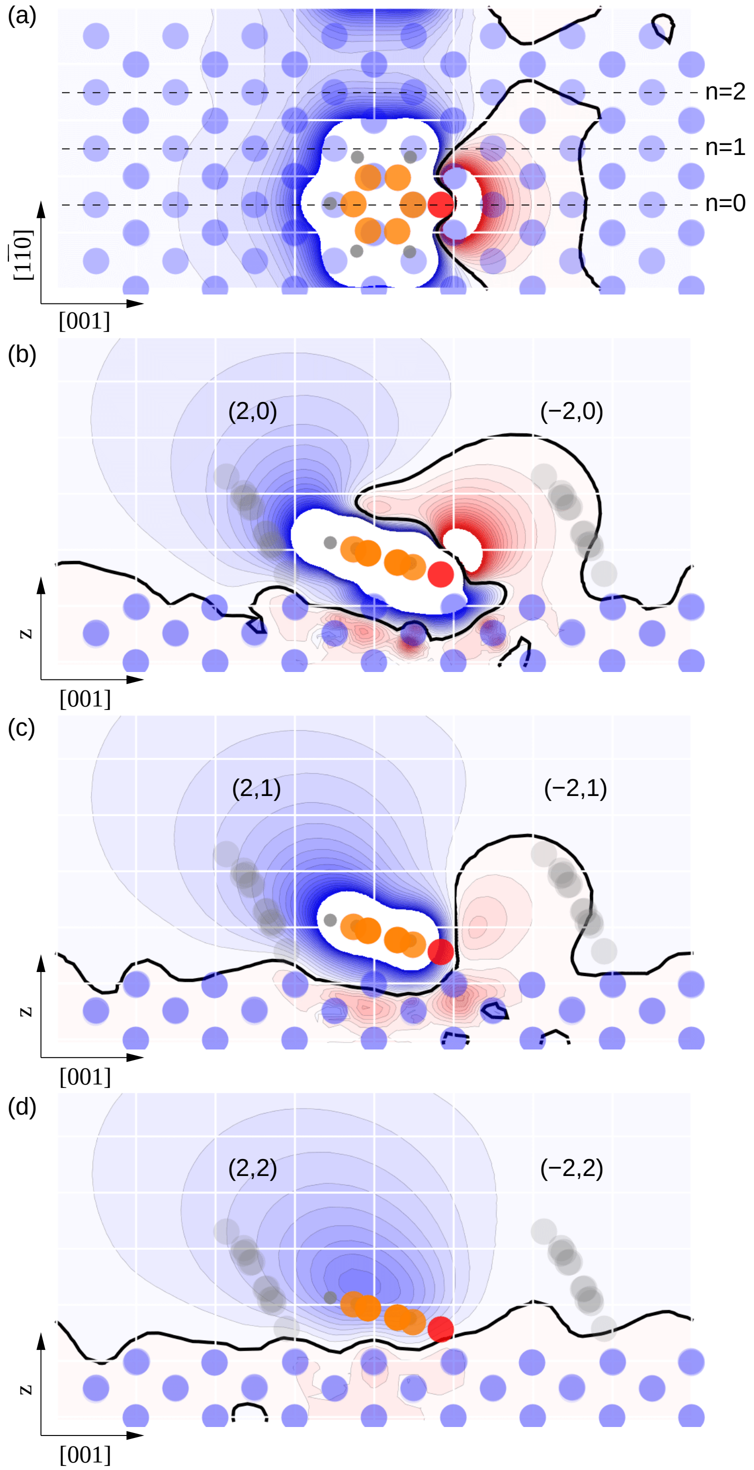

FIG. S7. **The change in the elecrostatic potential (δV) induced by a phenoxy molecule in a 8×5 representation of Cu(110).** (a) xy-plane with dashed lines corresponding to three xz-planes separated by 2.56 Å (b,c,d). Gray circles guide the eye to possible lifted phenoxy sites (not included in calculations). Contour lines are separated by 25 meV. Blue (red) areas indicate a postive (negative) change in potential and the thick contour line the zero-value. Atomic positions are shown as colored circles (blue: Cu, gray: H, orange: C, red: O). White lines indicate the Cu(110) lattice.

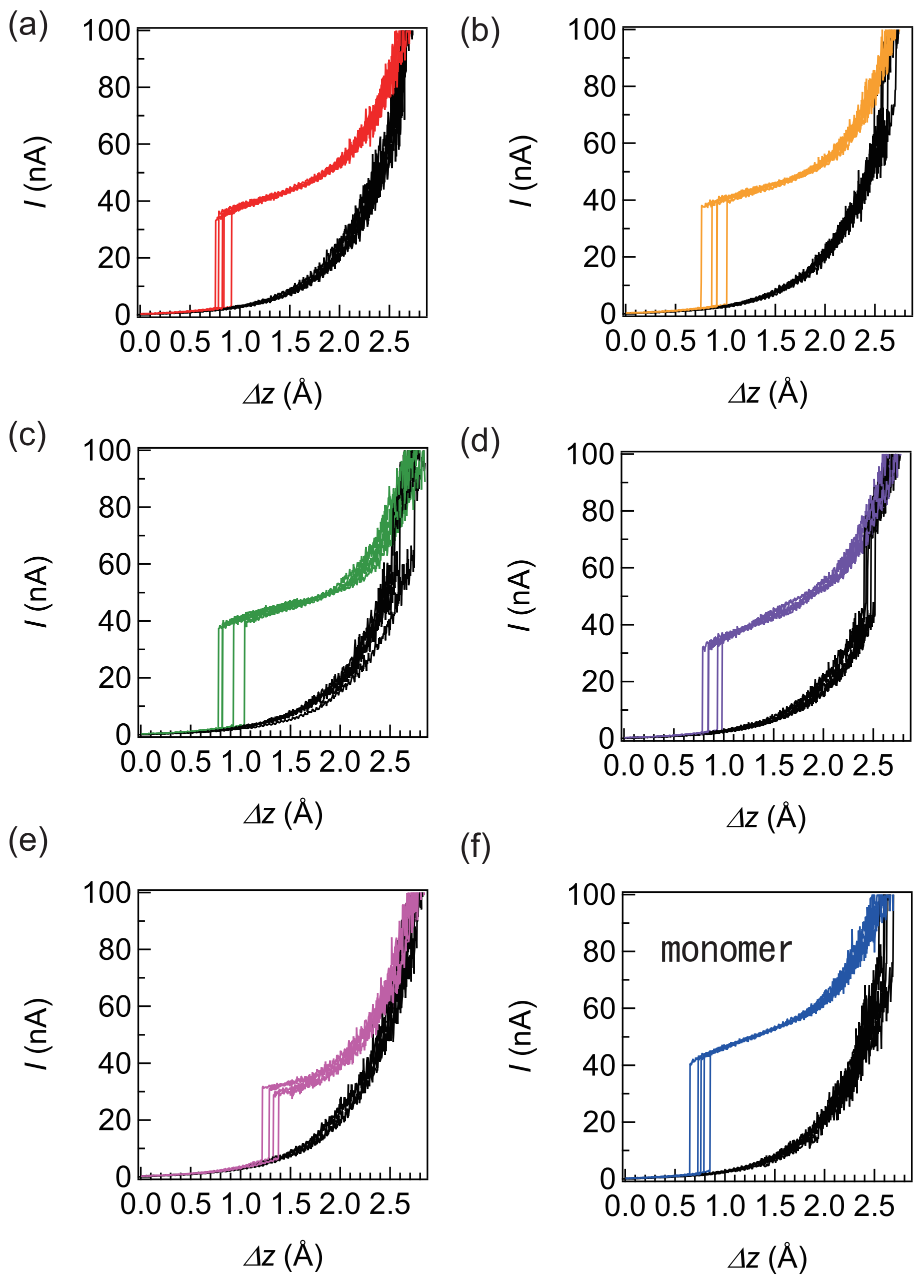

FIG. S8. **The I-Δz data for the island** The I-Δz curves obtained for each configuration in Figs. 7a-e [(a)-(e)], together with that for the monomer [(f)]. The curves were recorded by five cycles for each configuration, from which the molecular conductance was deduced (Fig. 7h).

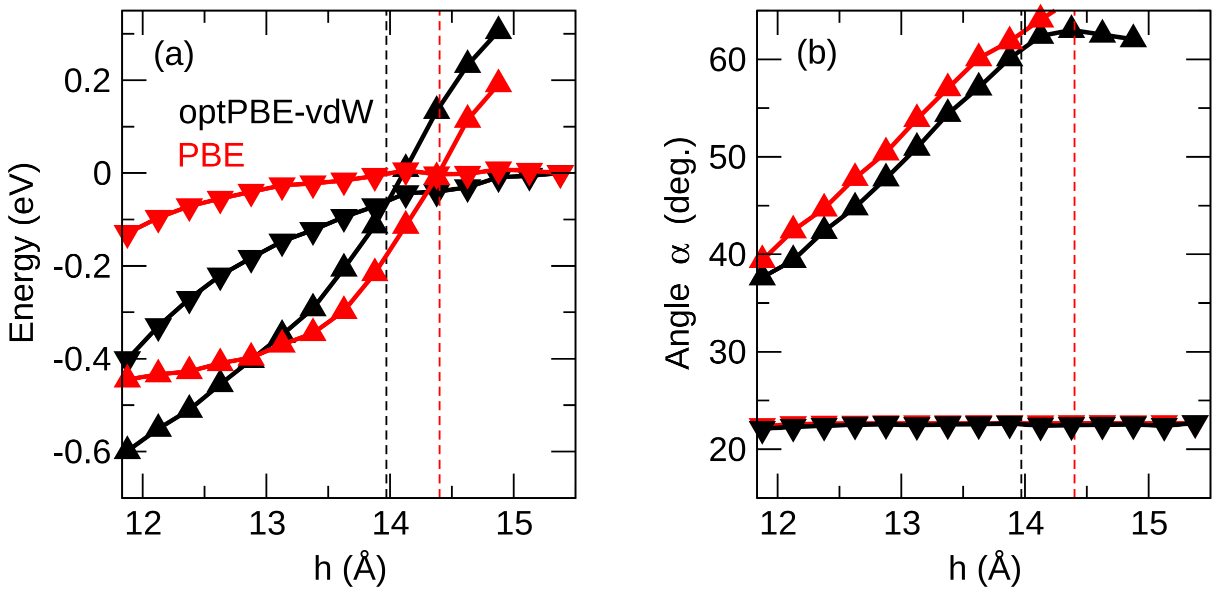

FIG. S9. **Comparison between optPBE-vdW and PBE functionals for the structure and stability diagram for phenoxy switching.** (a) Total energy differences and (b) molecular tilt angle with the optPBE-vdW (black data) and the PBE functional (red data) yield qualitatively the same results for the molecular switching. Tip lateral position is set to $x = 1.4$ Å (down-triangles: molecule flat, up-triangles: molecule lifted up). Vertical dashed lines indicate the tip height where flat and lifted configurations are energetically equal.

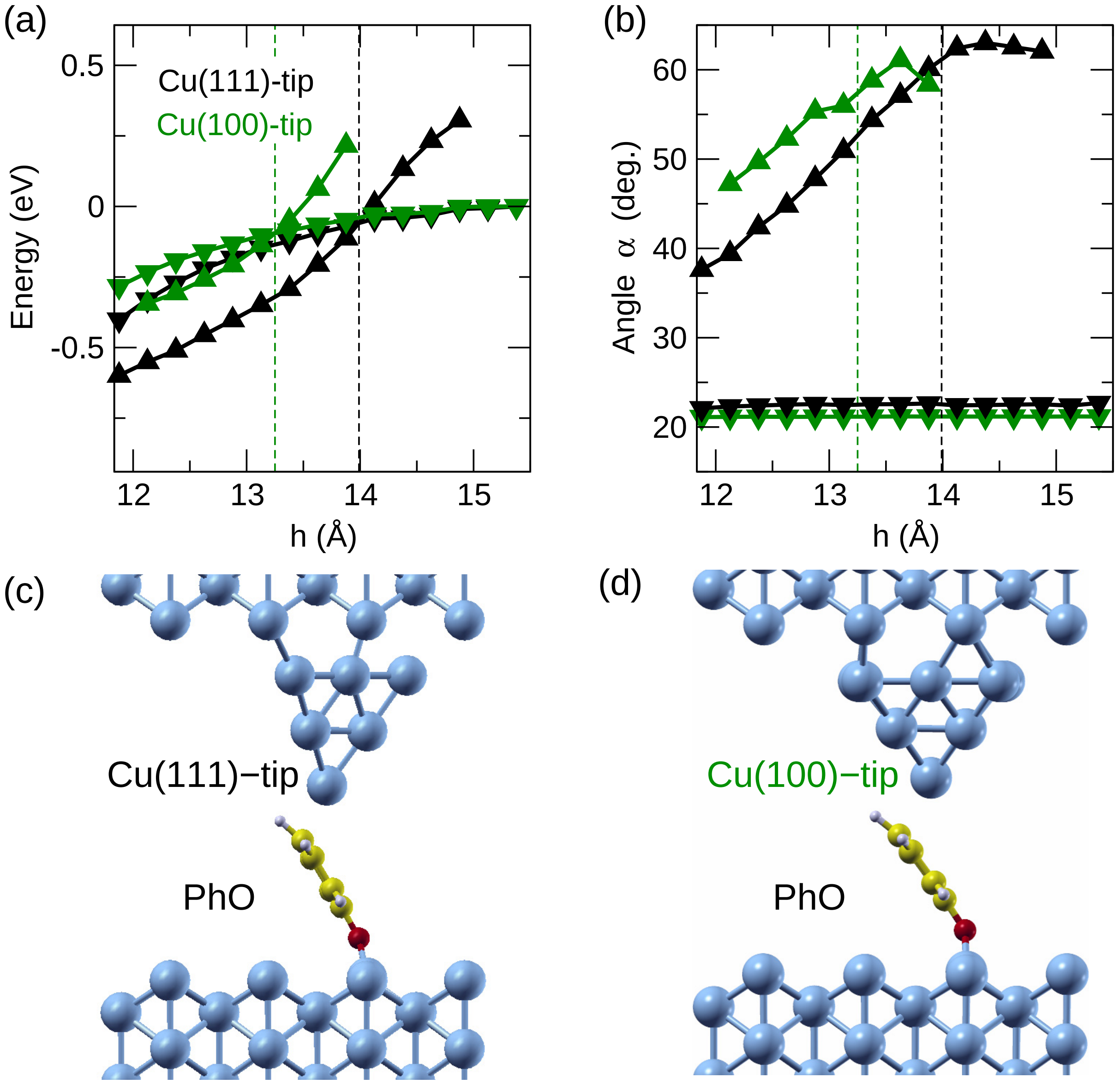

FIG. S10. **Comparison between Cu(111) and Cu(100) tip models for the structure and stability diagram for phenoxy switching.** (a) Total energy differences and (b) molecular tilt angle with the optPBE-vdW functional as function of tip height with lateral position fixed at $x = 1.4$ Å [black: Cu(111)-tip; green: Cu(100)-tip; down-triangles: molecule flat, up-triangles: molecule lifted up]. Vertical dashed lines indicate the tip height where flat and lifted configurations are energetically equal. (c) Lifted geometry of a phenoxy molecule in contact with the Cu(111) tip ($h = 13.9$ Å) and (d) the Cu(100) tip ($h = 13.1$ Å).

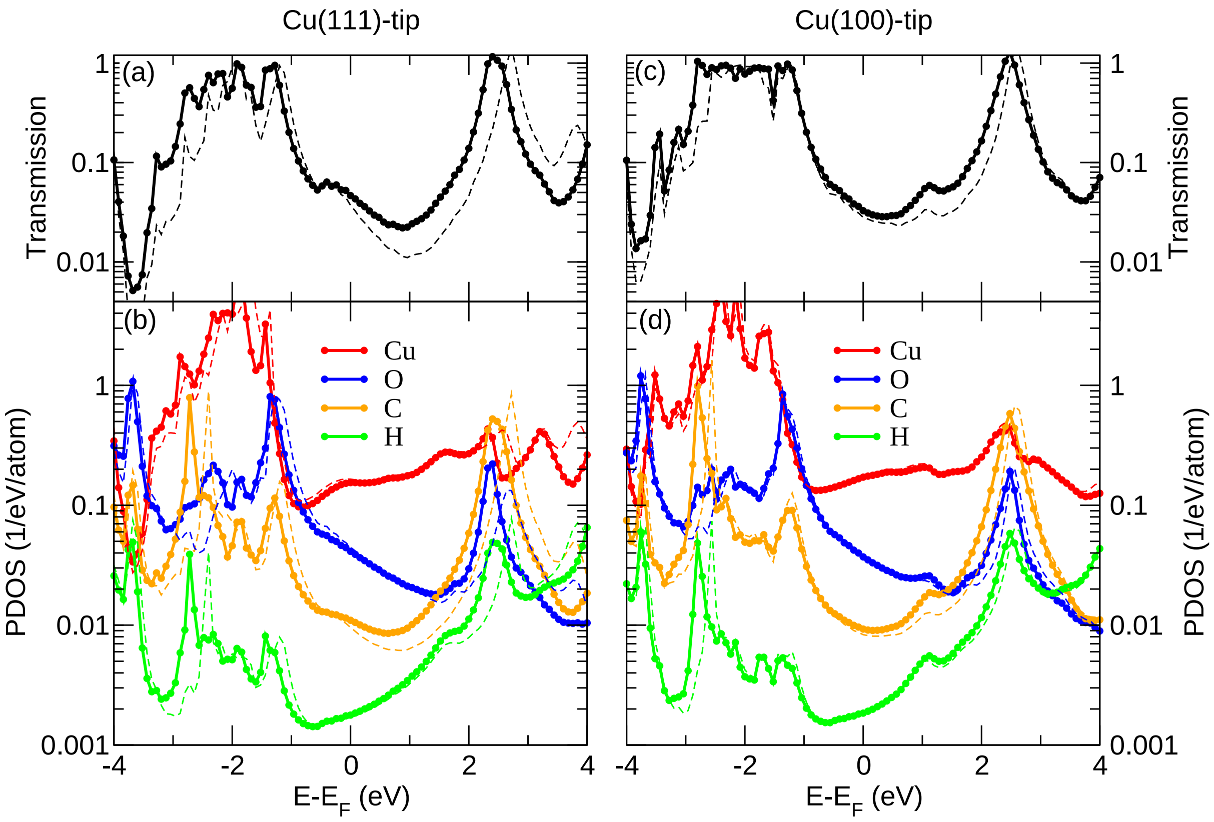

FIG. S11. **Electronic structure and electron transmission of phenoxy monomers contacted with Cu(111)- and Cu(100)-tips.** (a) Transmission and (b) projected density of states (PDOS) onto the Cu apex atom as well as onto the atoms in the phenoxy molecule with a Cu(111)-tip, cf. Fig. S10(c). Two different tip height are considered: $h = 13.9$ Å (full lines) and $h = 14.4$ Å (dashed lines). The lateral position is $x = 1.4$ Å. (c)-(d) Similar results for phenoxy contacted with a Cu(100)-tip, cf. Fig. S10(d): $h = 13.1$ Å (full lines) and $h = 13.6$ Å (dashed lines). In all cases the molecules are in their lifted configurations and the tip is laterally positioned at $x = 1.4$ Å.
